# Extreme Heat Exposure Is Associated with Higher Socioeconomic Disadvantage and Elevated Youth Delinquency

**DOI:** 10.31586/jsmhes.2024.1044

**Published:** 2024-08-18

**Authors:** Shervin Assari, Hossein Zare

**Affiliations:** 1 Department of Internal Medicine, Charles R. Drew University of Medicine and Science, Los Angeles, CA, United States; 2 Department of Family Medicine, Charles R. Drew University of Medicine and Science, Los Angeles, CA, United States; 3 Department of Urban Public Health, Charles R. Drew University of Medicine and Science, Los Angeles, CA, United States; 4 Marginalization-Related Diminished Returns (MDRs) Center, Los Angeles, CA, United States; 5 Department of Health Policy and Management, Johns Hopkins Bloomberg School of Public Health, Baltimore, MD, United States; 6 School of Business, University of Maryland Global Campus (UMGC), College Park, United States

**Keywords:** Extreme Heat, Climate Change, Delinquency, Child Development, Socioeconomic Status, Racial Disparities, Vulnerable Populations

## Abstract

**Background:**

Climate change has led to an increase in the frequency and intensity of extreme heat events, a trend expected to continue. This poses significant health risks, particularly for vulnerable populations like children. While previous research has largely concentrated on the physical health impacts of extreme heat, less attention has been given to behavioral outcomes, such as delinquency.

**Objectives:**

This study investigates the association between extreme heat exposure and delinquency among children, utilizing data from the Adolescent Brain Cognitive Development (ABCD) study. It also explores the potential mediating roles of neighborhood socioeconomic status (SES; measured by median home value), puberty, peer deviance, and financial difficulties.

**Methods:**

Data from the national ABCD study were analyzed to assess the relationship between extreme heat exposure (exposure) and delinquency (outcome). Covariates included race/ethnicity, sex, and age. Mediators examined were neighborhood SES, puberty, peer deviance, and financial difficulties. Structural equation modeling (SEM) was employed for data analysis.

**Results:**

Overall, 11,878 children entered our analysis. The analysis revealed a significant association between extreme heat exposure and higher levels of delinquency among children. Children more exposed to extreme heat were more likely to be Black, reside in lower SES neighborhoods, experience greater financial difficulties, and have more advanced puberty status. The group facing the highest heat exposure was also economically disadvantaged.

**Conclusions:**

The findings suggest that children already disadvantaged by socio-economic factors are disproportionately affected by extreme heat, leading to increased delinquency. This highlights the need for targeted interventions to protect these vulnerable populations and address the mediators of extreme heat exposure. Future research should focus on longitudinal studies and evaluate the effectiveness of various mitigation strategies to address these disparities.

## 1. Introduction

Global temperatures have surged to their highest levels in the past decade, marking unprecedented climatic changes since the 1850s. The Intergovernmental Panel on Climate Change (IPCC) [1] forecasts that the frequency and intensity of extreme temperatures will continue to rise, largely driven by climate change. This escalating trend has heightened concerns about the broad-ranging impacts of extreme weather, particularly in low-income areas, where resources to cope with such environmental challenges are often limited [2]. Economically, extreme weather events have been shown to escalate firms’ costs, reduce local demand, diminish agricultural yields, lower labor productivity, increase absenteeism, reduce local spending, and raise operational costs when adaptations are necessary [3–6].

The health ramifications of extreme heat are equally alarming [7, 8]. Hot ambient conditions and the associated heat stress can significantly elevate mortality and morbidity rates, adversely affect pregnancy outcomes, and negatively impact mental health. High heat stress can diminish physical work capacity and motor-cognitive performance, thereby affecting productivity and increasing the risk of occupational health problems [2]. Approximately half of the global population, including more than 1 billion workers, are exposed to high heat episodes, with about a third experiencing negative health effects [2]. However, many of these heat-related health risks are preventable through appropriate heat action plans that incorporate behavioral strategies and biophysical solutions [2]. The permanence of extreme heat events during summer seasons worldwide is causing numerous excess deaths, a trend projected to worsen with ongoing climate change. Particularly in tropical regions, increased warming may regularly push physiological limits related to heat tolerance, posing significant survival risks in the coming decades [2]. The interplay between climate change and other trends such as population growth, aging, urbanization, and socioeconomic development can further exacerbate or mitigate heat-related hazards. Urban areas, in particular, face enhanced temperatures due to anthropogenic heat from vehicular transport and heat waste from buildings [2].

Children are particularly vulnerable to the adverse effects of extreme heat. Evidence suggests that children aged 0-4 years face an increased risk of emergency department visits correlated with higher maximum temperatures (Tmax) on lag days 0, 1, and 3, with the strongest association observed on lag day 0. For instance, an increase in Tmax of 13°F resulted in an excess risk of 2.6% (95% CI: 2.2-3.0) for emergency visits. Age-stratified analyses reveal significant positive associations for same-day exposures, particularly among 1-4-year-olds, while children under 1 year of age show significant associations only on lag day 3. These patterns are consistent across various race/ethnicity subgroups and diagnostic categories, with notable excess risks for heat-specific diagnoses (16.6% [95% CI: 3.0-31.9]), general symptoms (10.1% [95% CI: 8.2-11.9]), infectious diseases (4.9% [95% CI: 3.9-5.9]), and injuries (5.1% [95% CI: 3.8-6.4]) [9, 10].

The Centers for Disease Control and Prevention (CDC) highlight that youth and children are among the vulnerable groups most at risk from extreme heat exposure. Extreme heat can disrupt children’s activities, such as play and socialization, leading to reduced community presence and altered social relationships. These effects are more pronounced for children living in poverty, who may lack access to mitigating resources like air conditioning. Despite these significant impacts, the literature on the influence of climate change and extreme heat exposure on youth development remains sparse. A 2014 systematic review [10] indicated that while mortality among children does not consistently rise during heat waves, infants are particularly susceptible to heat-related deaths. Pediatric conditions associated with heat waves include renal and respiratory diseases, electrolyte imbalances, and fever. Future research should focus on developing consistent definitions of heat waves from a children’s health perspective, identifying optimal measures of children’s heat exposure, and exploring sensitive outcome measures to quantify heat waves’ impacts on children. Moreover, understanding the varying vulnerabilities among children of different ages and socioeconomic backgrounds, projecting disease burdens under climate change scenarios, and developing effective mitigation and adaptation strategies are crucial [11].

Thus; as climate change continues to escalate the occurrence of extreme heat events [12–15], understanding their impact on child development, particularly concerning high-risk behaviors, becomes increasingly critical. This paper aims to fill this research gap by examining the relationship between extreme heat exposure and high-risk behaviors in children, leveraging data from the Adolescent Brain Cognitive Development (ABCD) [16–25] study. Given the role of neighborhood socioeconomic status (SES) [26, 27], financial difficulty [27], peer deviance [28], and puberty [29] as potential mediators, we investigated whether heat exposure is associated with youth delinquency through these constructs.

## 2. Methods

### 2.1. Design and Sample

We conducted a secondary analysis using data from the ABCD study [16–25], a national longitudinal study of a racially and economically diverse cohort of pre-adolescent children. The ABCD study’s methodology has been thoroughly documented elsewhere. Advantages of the ABCD dataset include its longitudinal design, national scope, large and diverse samples in terms of race, SES, and geographic distribution. Participants were primarily recruited from schools.

### 2.2. Analytical Sample

The analytical sample consisted of any ABCD youth regardless of their economic or race/ethnic background. Participants were 9–10-year-old at baseline. A total number of 11,878 youth entered our analysis.

### 2.3. Ethics

The study was approved by the Institutional Review Board (IRB) of the University of California, San Diego (UCSD). Assent was obtained from all participating adolescents, and informed consent was obtained from their parents.

### 2.4. Study Variables

The study variables included race/ethnicity, demographic and socioeconomic factors, adversities, and substance use.

#### Race/Ethnicity:

Parents reported the race and ethnicity of their children. This was a categorical variable with non-Latino White as the reference category.

#### Financial Stress:

Financial difficulties were assessed through financial difficulties experienced in the past 12 months. Items included inability to afford food, telephone service, rent/mortgage, eviction, utility shutoffs, and unmet medical or dental needs. Responses were binary (0 = no, 1 = yes), and a mean score was calculated, with higher scores indicating higher financial stress.

#### Delinquent Behaviors:

Rule-breaking and other forms of delinquency were self-reported by the children regarding their own behavior. These behaviors were measured on a continuous scale, with higher scores indicating greater involvement in delinquent activities. This measure served as the outcome variable, capturing the child’s engagement in risky behaviors.

#### Peer Delinquency:

Peer deviance was assessed through self-reports in which youth were asked about the high-risk behaviors and delinquency of their peers. This variable was measured on a continuous scale, with higher scores indicating greater levels of peer deviance. The measure captured the extent to which participants were exposed to peers engaging in behaviors such as substance use, rule-breaking, and other forms of delinquency as a risk factor of child behavior.

#### Puberty:

Puberty status was measured as a dichotomous variable, with participants categorized as either “any puberty” (coded as 1) or “pre-puberty” (coded as 0). This classification was based on the Tanner Stages, which assess pubertal development through observable body changes. For girls, this included indicators such as breast development, while for boys, it included the presence of facial and groin hair. This approach allowed for a clear distinction between children who had begun puberty and those who had not, providing a standardized measure of pubertal progression.

#### Neighborhood Median Home Value:

Using zip code data, the ABCD residential history provided a range of economic indicators for the residential area. Our variable represented a continuous measure of the median home value within the zip code, with higher values indicating a higher area-level SES.

### 2.5. Data Analysis

Data analysis was conducted using Stata. Univariate analysis involved reporting the mean and standard deviation (SD) of continuous measures. We used Pearson test to estimate bivariate correlations. Structural equation models (SEM) were used for multivariable analysis, with delinquency as the outcome. Predictor included heat wave exposure (state-level). Mediators were neighborhood SES, financial difficulties, puberty, or peer deviance. Age and gender were confounders. Collinearity between variables was checked and ruled out (all correlations were below 0.6). Beta (standardized path coefficient), 95% confidence intervals (CI), and p-values were reported.

## 3. Results

Table 1 presents the correlations between extreme heat exposure and various socio-economic and demographic factors. Extreme heat exposure has a weak negative correlation with neighborhood median income (r = −0.098), suggesting that low SES areas experience higher extreme heat exposure. This relationship is statistically significant, with a p-value of < 0.001. Extreme heat exposure is positively correlated with financial difficulties (r = 0.109), suggesting that individuals exposed to more extreme heat exposure experience financial difficulties, with a significant p-value of < 0.001. The influence of race/ethnicity on extreme heat exposure is varied. For example, being Black (r = 0.082) was associated with higher exposed to extreme heat with a significant p-value of < 0.001. Racial/ethnic categories such as White, Asian, and Other did not show correlations with extreme heat exposure, with p-values not indicating statistical significance. Additionally, the table indicates moderate correlations between extreme heat exposure and peer deviance (r = 0.042) and youth delinquency (r = 0.057). These correlations are significant with p-values of 0.010 and 0.006, respectively. These bivariate analyses revealed that exposure to extreme heat is significantly associated with being Black, living in lower SES families, residing in lower SES neighborhoods, and facing higher financial difficulties. Thus, the group experiencing the maximum heat exposure is already at an economic disadvantage.

**Figure 1.**
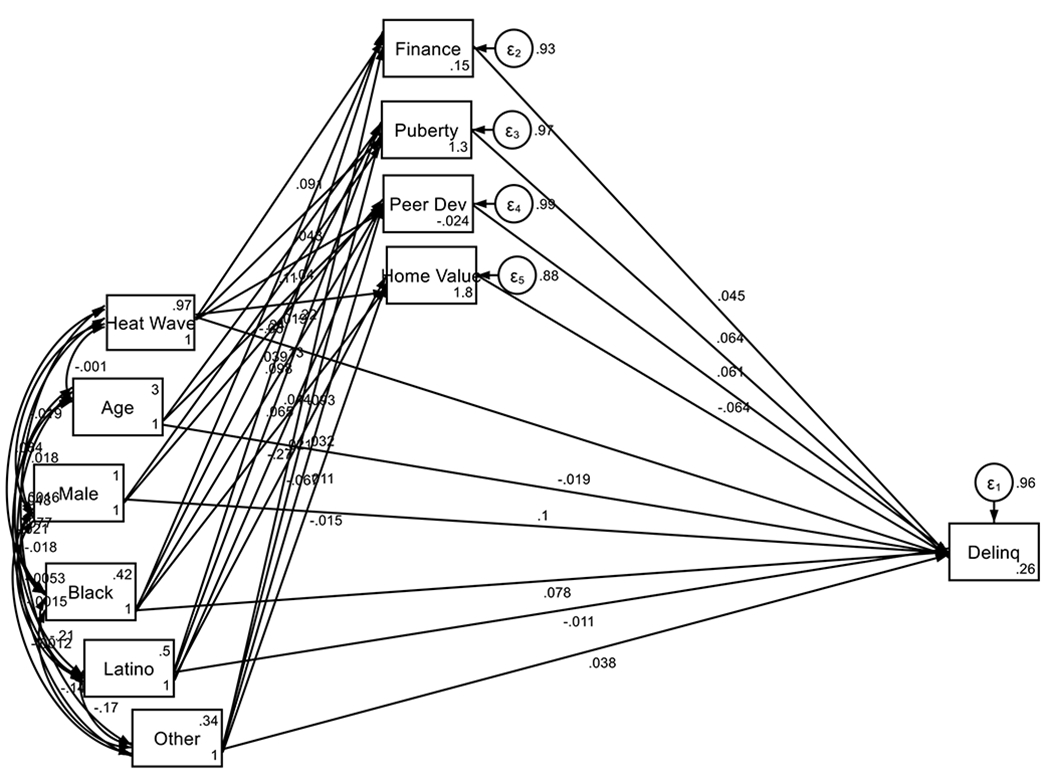
Summary of SEM

As shown by Table 2 and Figure 1, the analysis revealed that children who are more exposed to extreme heat are more likely to reside in lower SES neighborhoods, face higher financial difficulties, more advanced puberty, and report higher peer deviance. These variables are all associated with higher child delinquency. To be more specific, the findings show that extreme heat exposure is significantly linked to higher financial difficulties, with a beta of 0.09 (p < 0.001), indicating a moderate positive effect. Additionally, heat exposure has a direct, albeit smaller, effect on puberty (beta = 0.04; p < 0.001), pointing to a modest association between extreme heat and pubertal development. Black individuals show a significant positive effect on financial difficulties (beta = 0.24; p < 0.001), indicating that Black individuals are more affected by financial strains. Latino and Other racial/ethnic groups also show significant associations with financial difficulties, with betas of 0.13 (p = 0.000) and 0.09 (p = 0.000) respectively. When it comes to puberty, Black ethnicity shows a positive effect (beta = 0.10; p < 0.001), while Latino ethnicity shows a smaller effect (beta = 0.04; p < 0.001). Examining peer deviance and median home value, extreme heat exposure has a small positive impact on peer deviance (beta = 0.04; p = 0.015), suggesting a modest increase in deviant behaviors among peers in areas with extreme heat. Median home value, on the other hand, is lower in areas with extreme heat exposure (beta = −0.22; p < 0.001), indicating that higher extreme heat exposure is associated with lower home values. Financial difficulties and other factors such as pubertal status, peer deviance, and median home value have significant impacts on youth delinquency. Financial difficulties have a small but significant effect on delinquency (beta = 0.04; p = 0.053), while pubertal status and peer deviance have more pronounced effects, with betas of 0.06 (p = 0.002) and 0.06 (p = 0.001), respectively. Median home value shows a negative effect on delinquency (beta = −0.06; p = 0.002), indicating that lower home values are associated with higher delinquency.

## 4. Discussion

The primary aim of this study was to explore the association between exposure to extreme heat and delinquency among children and adolescents using data from the ABCD study [16–25]. Additionally, we sought to identify and understand the socio-demographic characteristics that may exacerbate the vulnerability of certain groups of children to the adverse effects of extreme heat, particularly focusing on race/ethnicity, SES of families and neighborhoods, and financial difficulties. Finally, the study aimed to explore the behavioral health implications of extreme heat exposure in terms of individual delinquency.

Our analysis of a national sample from the ABCD study revealed that exposure to extreme heat is significantly associated with being Black, living in lower SES families, residing in lower SES neighborhoods, and facing higher financial difficulties. Furthermore, exposure to extreme heat is linked to higher levels of delinquency, both individually and among peers. These findings suggest that the most vulnerable groups of children, who are already disadvantaged by various socio-economic factors, are also the ones experiencing the maximum exposure to extreme heat, thereby compounding their risk of adverse behavioral outcomes.

For Black children, the increased exposure to extreme heat can be understood through several mechanisms. The legacy of slavery has resulted in a higher concentration of Black populations in southern states, which experience more heat waves [30–32]. These states also have higher poverty rates [33, 34]. Systemic racism and historical residential segregation have led to Black communities often residing in neighborhoods with fewer resources to combat extreme heat, such as air conditioning, cooling centers, and shaded areas [35]. Additionally, Black communities frequently live in urban areas with high population density, pollution, heat-generating industries, and limited green spaces—all factors contributing to the urban heat island effect [36, 37]. This phenomenon exacerbates heat exposure, which is likely to worsen, especially during heat waves.

Children from lower SES families are more likely to be exposed to extreme heat due to limited access to resources that could mitigate these conditions [38]. Low SES families often live in substandard housing with inadequate insulation and cooling systems [39]. They are less likely to have well-functioning air conditioning, which is expensive, uses high electricity, and requires high maintenance [40–42]. The financial constraints in low SES populations make it challenging to afford air conditioning or even fans, increasing their susceptibility to heat-related stress [43]. The added strain of financial insecurity can also lead to higher stress levels, contributing to behavioral issues such as delinquency.

Neighborhoods with lower SES are often characterized by poor infrastructure, lack of green spaces, and higher levels of pollution, all of which can amplify the effects of extreme heat [44]. These neighborhoods are also less likely to have air-conditioned homes. These neighborhoods may lack community resources such as public pools, cooling centers, and parks, which can provide relief during extreme heat events. The cumulative stress of living in such environments can lead to increased behavioral problems among children.

Families experiencing higher financial difficulties are more likely to be exposed to heat waves. Families experiencing higher financial difficulties are less likely to have the means to buffer against extreme heat [45, 46]. Financial stress can exacerbate the effects of heat exposure by limiting the ability to afford cooling solutions or move to cooler environments [43, 47].

The study found that exposure to extreme heat is associated with increased individual delinquency among children. This can be attributed to several factors, including physiological stress responses to heat, which can impair cognitive functioning and self-regulation. Mental health may also be worse under heat extremes. Additionally, the discomfort and irritability caused by extreme heat can lead to aggressive behavior and poor decision-making, contributing to delinquent acts. The chronic stress can affect parenting practices, leading to less supervision and support for children, which can, in turn, lead to higher rates of delinquency.

Peer deviance was also found to be higher among children exposed to extreme heat. This may be a mechanism why we observe high delinquency in children exposed to heat waves. This can be explained by the increased likelihood of children congregating in outdoor spaces during heatwaves, often unsupervised, leading to group behaviors that can escalate into delinquency. Furthermore, children from disadvantaged backgrounds may lack access to organized activities and safe indoor spaces, making them more susceptible to engaging in delinquent behavior with peers.

### 4.1. Implications

The findings of this study have significant implications for public health and social policy. First, there is a need for targeted interventions to protect vulnerable populations from the adverse effects of extreme heat. This includes improving housing conditions, increasing access to cooling centers, and implementing community-based programs to provide safe activities for children during extreme heat events. Schools and local governments should also be aware of the heightened risks and take steps to ensure that children have access to air-conditioned environments and are not exposed to extreme heat during school hours or extracurricular activities.

### 4.2. Future Research

Future research should focus on long-term studies that compare decades of data to better understand the long-term impacts of extreme heat exposure on child development and delinquency. Such long-term studies would allow for the tracking of individual children over time, providing more robust data on how continuous exposure to extreme heat affects behavioral outcomes and developmental trajectories. Additionally, future research should examine the cumulative effects of multiple heat waves over the course of a child’s development, considering the potential for chronic stress and its implications on mental health and behavior. It is also important to explore the interaction between extreme heat exposure and other environmental and social stressors, such as air pollution and community violence, to gain a more comprehensive understanding of the multifaceted impacts on child development. Furthermore, investigating the differential impacts of heat exposure across various geographic regions and climates can provide insights into regional vulnerabilities and help tailor interventions accordingly.

Research should also explore the effectiveness of various interventions aimed at mitigating the effects of extreme heat on vulnerable populations. This includes evaluating the impact of community cooling centers, urban greening initiatives, and housing improvements on reducing heat exposure and its associated health and behavioral outcomes. Studies should assess the accessibility and utilization of these interventions by different demographic groups to identify potential barriers and inform more inclusive and effective policy designs. Additionally, there is a need to investigate the role of schools and educational programs in educating children and families about heat safety practices and promoting behaviors that can reduce heat-related risks. Understanding the cost-effectiveness and feasibility of implementing these interventions at a larger scale can aid policymakers in making informed decisions to protect vulnerable populations.

### 4.3. Limitations

This study has several limitations that should be acknowledged. One significant limitation is the reliance on self-reported data for delinquency, which may be subject to reporting bias. Children and adolescents may underreport their delinquent behaviors due to social desirability. Any self-reported measure is prone to recall biases. To mitigate this, future studies could incorporate objective measures of delinquency, such as school disciplinary records or official crime reports, to provide a more accurate assessment of delinquent behavior. Another limitation is the cross-sectional nature of the data, which restricts the ability to draw causal inferences. While the associations between extreme heat exposure and delinquency are evident, the cross-sectional design does not allow for the determination of temporal relationships or the directionality of these associations.

Additionally, the study may not fully account for all potential confounding variables that could influence the observed relationships. Factors such as parental supervision, access to recreational facilities, and individual temperament could also play significant roles in mediating the impact of extreme heat on delinquency. Future research should aim to include a more comprehensive set of control variables to isolate the specific effects of heat exposure. The generalizability of the findings may also be limited by the specific demographic and geographic characteristics of the sample. Despite these limitations, the study provides valuable insights into the link between extreme heat exposure and delinquency among children and highlights the need for targeted interventions to mitigate these adverse effects. ABCD is conducted in diverse populations and different regions that can increase the validity of the observed findings and ensure their applicability to broader contexts.

## 5. Conclusion

In conclusion, this study highlights the significant association between extreme heat exposure and increased delinquency among children. We also observed that the same population is more likely to be Black, lower SES families and neighborhoods, and those facing higher financial difficulties. The findings underscore the urgent need for targeted interventions and policies to mitigate the adverse effects of extreme heat on vulnerable populations and improve their overall well-being and developmental outcomes. As climate change continues to exacerbate extreme heat events, addressing these disparities becomes increasingly critical to safeguarding the health and development of all children.

## Figures and Tables

**Table 1.** Correlations Between Study Variables

	1	2	3	4	5	6	7	8	9	10	11	12
1 Extreme Heat Exposure	1											
												
												
2 Neighborhood Median Income / 5000	−0.098	1										
	< 0.001											
												
3 Neighborhood Poverty Status	0.046	−0.764	1									
	< 0.001	< 0.001										
												
4 Puberty Status	0.054	−0.059	0.055	1								
	< 0.001	< 0.001	< 0.001									
												
5 Financial Difficulties	0.109	−0.225	0.248	0.038	1							
	< 0.001	< 0.001	< 0.001	< 0.001								
												
6 Race/Ethnicity (White)	−0.064	0.366	−0.400	−0.084	−0.206	1						
	< 0.001	< 0.001	< 0.001	< 0.001	< 0.001							
												
7 Race/Ethnicity (Black)	0.082	−0.315	0.373	0.090	0.206	−0.098	1					
	< 0.001	< 0.001	< 0.001	< 0.001	< 0.001	< 0.001						
												
8 Race / Ethnicity (Latino)	−0.046	−0.195	0.192	0.013	0.055	0.046	−0.764	1				
	< 0.001	< 0.001	< 0.001	0.199	< 0.001	< 0.001	< 0.001					
												
9 Race/Ethnicity (Asian)	−0.015	0.053	−0.062	0.007	−0.047	0.054	−0.059	0.055	1			
	0.134	< 0.001	< 0.001	0.474	< 0.001	< 0.001	< 0.001	< 0.001				
												
10 Race/Ethnicity (Other)	0.076	0.002	−0.003	0.013	0.044	0.109	−0.225	0.248	0.038	1		
	< 0.001	0.809	0.733	0.202	< 0.001	< 0.001	< 0.001	< 0.001	< 0.001			
												
11 Peer Deviance	0.042	−0.046	0.055	0.041	0.066	−0.120	0.459	−0.549	−0.068	−0.424	1	
	0.010	0.004	0.001	0.014	< 0.001	< 0.001	< 0.001	< 0.001	< 0.001	< 0.001		
												
12 Youth Delinquency	0.057	−0.051	0.052	0.066	0.074	−0.073	0.384	−0.452	−0.055	−0.271	0.622	1
	0.006	0.012	0.011	0.002	< 0.001	< 0.001	< 0.001	< 0.001	< 0.001	< 0.001	< 0.001	

**Table 2.** Summary of the Structural Equation Model (SEM).

Predictor		Outcome	95%				
			Beta	SE	CI		P
Extreme Heat Exposure	→	Financial Difficulties	0.09	0.01	0.07	0.11	< 0.001

Race/Ethnicity (Latino)	→	Financial Difficulties	0.13	0.01	0.11	0.14	< 0.001

Race/Ethnicity (Black)	→	Financial Difficulties	0.24	0.01	0.22	0.26	< 0.001

Race/Ethnicity (Other)	→	Financial Difficulties	0.09	0.01	0.08	0.11	< 0.001

Intercept	→	Financial Difficulties	0.15	0.02	0.12	0.18	< 0.001

Extreme Heat Exposure	→	Puberty	0.04	0.01	0.02	0.06	< 0.001

Race/Ethnicity (Latino)	→	Puberty	0.04	0.01	0.02	0.06	< 0.001

Age (10)	→	Puberty	0.11	0.01	0.09	0.13	< 0.001

Sex (Male)	→	Puberty	−0.08	0.01	−0.10	−0.06	< 0.001

Race/Ethnicity (Black)	→	Puberty	0.10	0.01	0.08	0.12	< 0.001

Race/Ethnicity (Other)	→	Puberty	0.03	0.01	0.01	0.05	0.002

Intercept	→	Puberty	1.27	0.04	1.19	1.35	< 0.001

Extreme Heat Exposure	→	Peer Deviance	0.04	0.02	0.01	0.07	0.015

Race/Ethnicity (Latino)	→	Peer Deviance	0.02	0.02	−0.01	0.05	0.212

Age (10)	→	Peer Deviance	0.02	0.02	−0.01	0.05	0.246

Sex (Male)	→	Peer Deviance	0.04	0.02	0.01	0.07	0.013

Race/Ethnicity (Black)	→	Peer Deviance	0.06	0.02	0.02	0.10	0.001

Race/Ethnicity (Other)	→	Peer Deviance	0.01	0.02	−0.02	0.04	0.513

Intercept	→	Peer Deviance	−0.02	0.06	−0.14	0.09	0.685

Extreme Heat Exposure	→	Median Home Value	−0.22	0.01	−0.23	−0.20	< 0.001

Race/Ethnicity (Latino)	→	Median Home Value	−0.07	0.01	−0.09	−0.05	< 0.001

Race/Ethnicity (Black)	→	Median Home Value	−0.27	0.01	−0.28	−0.25	< 0.001

Race/Ethnicity (Other)	→	Median Home Value	−0.01	0.01	−0.03	0.00	0.114

Intercept	→	Median Home Value	1.77	0.02	1.74	1.80	< 0.001

Financial Difficulty	→	Delinquency	0.04	0.02	0.00	0.09	0.053

Puberty (Any)	→	Delinquency	0.06	0.02	0.02	0.11	0.002

Peer Deviance	→	Delinquency	0.06	0.02	0.03	0.10	0.001

Median Home Value/	→	Delinquency	−0.06	0.02	−0.10	−0.02	0.002

Race/Ethnicity (Latino)	→	Delinquency	−0.01	0.02	−0.05	0.03	0.594

Age (10)	→	Delinquency	−0.02	0.02	−0.06	0.02	0.345

Sex (Male)	→	Delinquency	0.10	0.02	0.06	0.14	< 0.001

Race/Ethnicity (Black)	→	Delinquency	0.08	0.03	0.03	0.13	0.002

Race/Ethnicity (Other)	→	Delinquency	0.04	0.02	0.00	0.08	0.083

Intercept	→	Delinquency	0.26	0.08	0.10	0.42	0.002
